# Functional Region Annotation of Liver CT Image Based on Vascular Tree

**DOI:** 10.1155/2016/5428737

**Published:** 2016-11-07

**Authors:** Yufei Chen, Xiaodong Yue, Caiming Zhong, Gang Wang

**Affiliations:** ^1^Research Center of CAD, Tongji University, Shanghai 200092, China; ^2^School of Computer Engineering and Science, Shanghai University, Shanghai 200444, China; ^3^College of Science and Technology, Ningbo University, Ningbo 315211, China; ^4^School of Statistics and Management, Shanghai University of Finance and Economics, Shanghai 200433, China

## Abstract

Anatomical analysis of liver region is critical in diagnosis and treatment of liver diseases. The reports of liver region annotation are helpful for doctors to precisely evaluate liver system. One of the challenging issues is to annotate the functional regions of liver through analyzing Computed Tomography (CT) images. In this paper, we propose a vessel-tree-based liver annotation method for CT images. The first step of the proposed annotation method is to extract the liver region including vessels and tumors from the CT scans. And then a 3-dimensional thinning algorithm is applied to obtain the spatial skeleton and geometric structure of liver vessels. With the vessel skeleton, the topology of portal veins is further formulated by a directed acyclic graph with geometrical attributes. Finally, based on the topological graph, a hierarchical vascular tree is constructed to divide the liver into eight segments according to Couinaud classification theory and thereby annotate the functional regions. Abundant experimental results demonstrate that the proposed method is effective for precise liver annotation and helpful to support liver disease diagnosis.

## 1. Introduction

As a noninvasive and painless medical test, Computed Tomography (CT) imaging can provide volumetric image data for liver disease diagnosis, which has been widely used in hospitals [1, 2]. The Computer Aided Diagnosis (CAD) on liver is a complex task that depends on a good understanding of the whole liver system, including the features of liver, vessels and lesions, as well as the anatomical features on specific patients [3–5]. Automatically annotating the functional segments of liver is an effective way to support doctors to study and precisely evaluate the liver system. Although there have been limited research works on liver annotation [6–8], its implementation for real CAD applications is still an arduous task for the following reasons.

The first and fundamental step in liver annotation is organ region segmentation [9]. Some methods segment organ through modeling the shape of liver region [10–13]. However, because of the large variance of liver shapes among different patients, it is difficult for shape-based methods to achieve precise liver segmentation. As another popular segmentation method, the level set methods are very sensitive to liver contour initialization and suffer from iterative computation burden [14–16]. In particular, for the images with tumors and vessels located near liver surface, the level-set-based segmentation tends to be trapped into local optima and eliminates tumors and vessels from main target. Recent research works reveal that the graph cut models have a great potential with the advantages of global optimization and practical efficiency for image segmentation [17]. But, for CT images of livers, the graph cut models cannot handle well seriously blurred boundaries and are incapable of distinguishing the liver regions of similar intensities from neighboring organs [18, 19].

Besides organ region segmentation, vessel segmentation has been another challenging task for liver annotation due to the limitation of vascular imaging equipment and the complexity of liver vascular topology. Florin et al. [20] treated the vessel segmentation as a tracking problem, where vessels were iteratively tracked using information on centerlines and local features. The method needs user interaction and requires special routines to handle branch points. Selle et al. [21] presented an intensity-threshold-based method, which defined an optimal intensity threshold through measuring region growing. This kind of methods mainly suits segmenting large vessels. Manniesing et al. proposed a vessel segmentation method based on Hessian matrix, which enhances liver vessels and thereby extracts tubular structures from the organ region [22]. The vesselness of the structure is determined by its geometrical features obtained from the eigensystem of Hessian matrix [23].

The topology of liver vasculature is of importance for recognizing functional segments. Vessel skeleton is widely used to present the topology of vasculature. Generally speaking, there are three types of vessel skeletonization methods. The algorithms based on distance transformation describe well the local structure but cannot guarantee the connectivity and completeness of skeleton [24]. The algorithms based on Voronoi diagram are capable of capturing the topology of the entire vasculature, but the computation of Voronoi skeleton is time-consuming, especially for the medical image containing large-size objects [25]. Compared with the skeletonization methods above, the thinning algorithms can extract the vessel skeleton efficiently and, in the meantime, preserve the connectivity and completeness of the skeleton. Moreover, the thinning algorithm can also record the medial position of the skeleton, which is very helpful to annotate the functional regions [2, 26]. However, only vessel skeletons without quantitative measurement are not sufficient to present the real topology of vasculature [27]. For example, it is difficult for vessel skeletons to keep the important organ features such as length, blood flow direction, and radius of subbranch. Without considering anatomical measurements, the imperfect vessel segmentation results may lead to cyclic skeleton.

**Figure 1:**
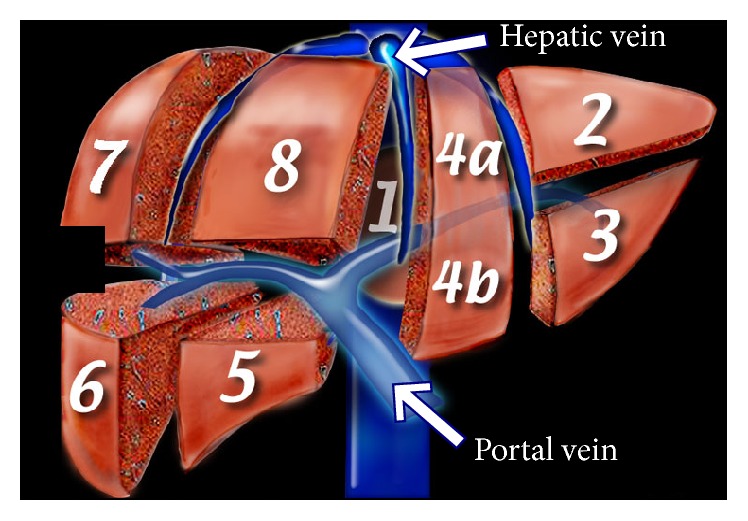
Couinaud classification of liver anatomy.

Obtaining the topology of liver vasculature, we can partition liver region into functional segments and make the annotation. Referring to the Couinaud classification of liver anatomy, hepatic and portal veins divide liver into eight functionally independent segments as shown in Figure 1 [28]. Each segment has its own vascular inflow, outflow, and biliary drainage. In the center of each segment, there is a branch of the portal vein, hepatic artery, and bile duct. In the periphery of each segment, there is vascular outflow through the hepatic veins. Middle hepatic vein divides the liver into right and left lobes. Right hepatic vein divides the right lobe into anterior and posterior segments. Left hepatic vein divides the left lobe into a medial and lateral part. Portal vein divides the liver into upper and lower segments.

Formulating the Couinaud classification of liver anatomy, Oliveira et al. estimated the planes that best fit each of the three branches of the hepatic veins and the plane that best fits the portal vein. These four planes define the subdivision of the liver in the Couinaud segments [29]. However, the plane-based method does not consider the influence of vascular variation to plane estimation, and it ignores the fact that the separation between liver segments should be a surface. Selle et al. segmented liver through computing the nearest neighbor of different vessel branches [21]. But the result depends on the user defined parameter, which should be manually adjusted under different cases. Schenk et al. used a Laplace model to assign each liver cell to one of the vascular branches to form liver segments [30]. This model suffers from a great computation burden and is not robust enough due to the dependence on vascular branch calibration. Huang et al. designed a fast liver segment method based on the hepatic vessel tree [31]. The method projects the liver and vessel tree to a plane and the classification of liver is achieved by classifying points in the projection plane. Although having high efficiency, the method based on hepatic vessel tree is not a complete functional anatomy, which leads to the inconsistency between the annotated liver segments and the actual blood-supply branch.

As mentioned above, the topology of vasculature can guide the annotation of functional segments. However, because of the high complexity of liver vasculature, it is hard to generate a precise representation of vasculature topology and the computation of geometric structures of all the vessels is always a time-consuming task. In fact, from the view of anatomy, the left and right portal vein branches superiorly and inferiorly project into the center of each segment to supply blood. This means that the functional segments of liver can be recognized by only portal vein branches in it. In the light of this finding, we proposed a hierarchical vascular tree to present the topology of portal veins. The branches of hierarchical vascular tree of portal veins correspond to the functional segments of liver. Based on the obtained functional segments, we annotate the liver region and measure the organ attributes using a standard terminology [6]. The visualization of annotation and the measurements can form a report of liver system for CAD. The contributions of this paper are summarized as follows.

*(1) Design a Vessel Tree to Present the Topology of Portal Veins*. Connect the topological voxels in vessel skeleton to form a graph. Prune redundant and irrelevant branches of graph to generate a formal vessel tree, which indicates the geometric structures of portal veins and blood flow direction.

*(2) Extend the Vessel Tree to a Hierarchical One for Liver Annotation*. The vessel tree is hierarchically divided into two levels according to the radius of portal vein branches. The Second Subtree branches are preserved to form functional segments.

This paper is organized as follows. The workflow of the proposed vessel-tree-based liver annotation method for CT images is described in Section 2.  Section 3 gives a detailed introduction of the proposed method, which includes vessel tree generation, liver segment, and annotation. In Section 4, abundant experimental results validate the effectiveness of the proposed annotation method. The paper work is summarized in Section 5.

## 2. Methodology

**Figure 2:**
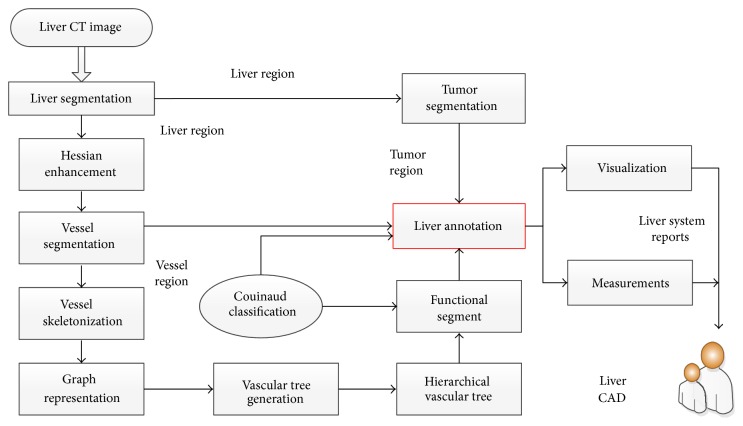
Workflow of the proposed annotation method.

In this section, we describe the entire workflow of the proposed functional region annotation method for liver CT images. The workflow consists of four stages. At the first stage, the liver region containing vessels and tumors is segmented from the CT image using an improved graph cut model. More details of liver region segmentation can be found in our previous work [32]. In the liver region, the segmentation for tumors and vessels is further performed. Second, a 3-dimensional thinning algorithm is applied to extract the skeleton of vessels from the segmented vessel region. Based on the skeleton, the topological structure of the vessel system is represented by a vascular tree. Specifically, the vascular tree is formulated by a directed acyclic graph and it can be further extended to a hierarchical version. The hierarchical vascular trees present the connectivity of vessels among the functional segments of liver. According to the connectivity of vascular trees, the liver region can be divided into eight functional segments referring to Couinaud classification theory. At the third stage, integrating the segmentation results of tumors, vessels, and functional segments, we can annotate the liver region and measure the attributes of organ. Finally, a report of liver system which includes the visualization of region annotation and the measurements of functional segments is generated to support doctors to precisely evaluate the liver system. The core steps of the workflow are illustrated in Figure 2; the details will be further introduced in the following section.

## 3. Vessel-Tree-Based Liver Annotation

As introduced above, the key step of liver annotation is to partition the liver region into multiple functional segments and the topology of vasculature can provide prior information to guide the partition. According to the Couinaud classification theory, the liver system can be divided into eight functional anatomies. It is not necessary to analyze the geometric structure of the entire vasculature; the partition can be performed through constructing a hierarchical vascular tree of portal veins. The methodologies of constructing trees of portal veins and annotating liver segments with hierarchical vessel trees will be elaborated in this section.

### 3.1. Vessel Tree Generation

#### 3.1.1. Vessel Segmentation

Vessel segmentation is a preliminary step for liver annotation. In this step, first, the liver segmentation is performed on CT image and then the vessels and tumors are further extracted from the segmented liver region. Precise segmentation of liver region is crucial to the subsequent annotation and measurement. In the proposed method, we adopt a semisupervised approach for liver segmentation of CT scans. The segmentation method is based on a graph cut model integrated with domain knowledge, which combines both boundary and regional cues in a global optimization framework. Specifically, the pixels in each CT scan are represented by a graph and the problem of region segmentation is casted to searching for the optimal cut on graph. The energy function of graph cut is constructed via knowledge-based similarity measure and hard constraints are defined to speed up the graph computation. More details of liver region segmentation can be found in our previous work [32]. We use the same segmentation method to obtain the tumor region.

Extracting vessels from liver region is a prerequisite for the geometrical and structural analysis of vasculature, which is very important for liver disease diagnosis. To segment the regions of vessels, we use Hessian-based filter to enhance the contrast of liver region *I*_liver_. The filter is good at searching for tubular-like structures. For discriminating tubular-like structures from blob-like and plate-like structures, the eigenvalues of Hessian matrix for filtering should satisfy condition *λ*_1_ ≈ 0, *λ*_2_ ≪ 0, *λ*_3_ ≪ 0 [22]. The vesselness measure of structures is defined as follows:

(1) Vesselλ=0if  λ2≥0  or  λ3≥01−e−Ra2/2α2·e−Rb2/2β2·1−e−Rc2/2c2otherwise,Ra=λ2λ3,Rb=λ1λ2·λ3,Rc=λ12+λ22+λ32,

where *α*, *β*, *c* are the parameters to control the sensitivity of measure. In the experiments, we set *α* = 0.3, *β* = 0.7, *c* = *I*_max_/2. Upper bound *I*_max_ corresponds to the brightest intensity value of vessels that can be empirically defined. After the Hessian filtering, a 3D region growing algorithm is utilized on the filtered liver region to segment the liver vasculature. Some morphological operations are adopted to fill small cavities, so as to make the vessel region continuous and smooth. The segmented vessel region consists of portal veins and hepatic veins. As introduced in Section 1, the portal veins are sufficient for distinguishing the functional segments of liver; thus we preserve the connected component of portal vein as binary image *I*_vessel_, in which 1 stands for pixels of vasculature and 0 represents the background.

#### 3.1.2. Vessel Skeletonization

To capture the topology of vasculature, first, we should extract the vessel skeleton from the segmented vessel region. Vessel skeletonization aims to reduce the foreground region of binary image *I*_vessel_ to a skeletal remnant. The skeletonization process should preserve the extent and connectivity of the original vessel region. To satisfy these requirements, we design a 3D thinning algorithm to extract the skeleton of vessels. The skeleton obtained through spatial thinning can preserve the geometric structure of the original vessel region, situate in the middle of *I*_vessel_, and be single-voxel wide. Moreover, the thinning-based skeletonization is robust to noisy voxels. The thinning algorithm is implemented through categorizing the voxels.

**Figure 3:**
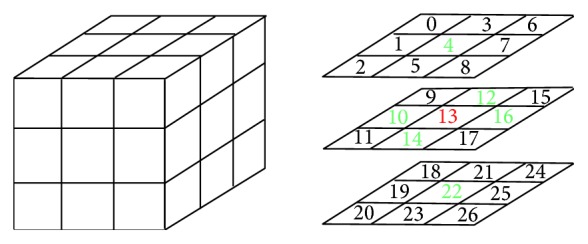
Neighborhood connectivity of a voxel.

In 3D space, a 3 × 3 × 3 lattice is built to examine the local connectivity of a voxel. The 26-neighborhood and 6-neighborhood (marked as green) connectivity is shown in Figure 3. Given voxel *v*, *N*_6_(*v*) and *N*_26_(*v*), respectively, denote the 6 neighbors and 26 neighbors of *v*. For skeletonization, the voxels can be categorized into four types: Border Voxel *V*_*B*_, Line Voxel *V*_*L*_, Euler Invariant Voxel *V*_*E*_, and Simple Voxel *V*_*S*_. Next, we expatiate the definitions of the voxels of different types.

**Definition 1 (Border Voxel).**

Given vessel voxel *v* ∈ *V*_vessel_, if at least one of its 6 neighbors has the value of 0, that is belonging to background, the voxel is considered as Border Voxel:

(2) VB=v ∣ NumberN6v=0≥1,  v∈Vvessel,Vvessel=v ∣ Ivesselv=1.

**Definition 2 (Line Voxel).**

Given vessel voxel *v* ∈ *V*_vessel_, if more than one of its 26 neighbors have the value of 1, that is belonging to vessels, the voxel is considered as Line Voxel:

(3) VL=v ∣ NumberN26v=1>1,  v∈Vvessel,Vvessel=v ∣ Ivesselv=1.

**Definition 3 (Euler Invariant Voxel).**

Given vessel voxel *v* ∈ *V*_vessel_, if Euler characteristic *χ* will not change when removing *v* from *V*_vessel_, the voxel is considered Euler Invariant:

(4) VE=v ∣ χVvessel∩N26v−χVvessel∩N26v∪v=0,  v∈Vvessel,χ=O−H+C,Vvessel=v ∣ Ivesselv=1,

where *O*, *H*, and *C* are, respectively, the numbers of connected objects, holes, and cavities in the image.

**Definition 4 (Simple Voxel).**

Given vessel voxel *v* ∈ *V*_vessel_, if the connectivity in its 26 neighborhoods keeps being invariant when removing *v* from *V*_vessel_, the voxel is considered as Simple Voxel:

(5) VS=v ∣ OVvessel∩N26v−OVvessel∩N26v∪v=0,  v∈Vvessel,Vvessel=v ∣ Ivesselv=1.

From the definitions above, we can find that the voxels of border and lines and Euler invariant and simple voxels are redundant for preserving the topology of vasculature. Thus, the skeletonization can be performed through iteratively deleting all those four kinds of voxels from vessel region, until no more change occurs. The output of skeletonization is binary image *I*_skeleton_ that contains a single-voxel wide skeleton marked as 1, noted as *V*_skeleton_.

#### 3.1.3. Graph Representation

To better understand the topology of vasculature, the structure of liver vessels represented by skeleton is further formulated by a graph. The graph consists of a set of vertexes (topological voxels) and connecting edges.

**Definition 5 (topological voxels).**

Topological voxels consist of end-voxels and branch-voxels: end-voxel is the voxel in *V*_skeleton_ with only one skeleton neighbor and branch-voxel is the skeleton voxel having more than two skeleton neighbors.

**Figure 4:**
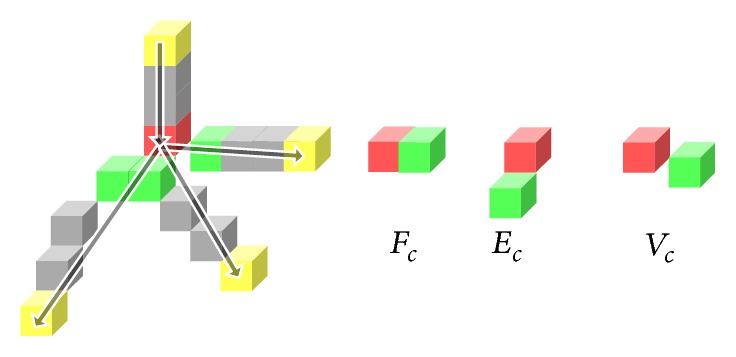
Topological voxels in vessel skeleton.

As shown in Figure 4, end-voxels can be easily found by counting the skeleton number in its 26 neighborhoods, which are marked as yellow. However, for branch-voxels, there are four candidates that have more than two neighbors (marked in red and green). Among all the possible branch-voxel candidates, the real branch-voxel should have the highest connectivity with all its neighboring branches, as the red voxels shown in Figure 4. The connectivity of neighboring branches can be quantified by the following cost function:

(6) vcandidate=v ∣ NumberN26v=1≥2,  v∈Vskeleton,costv=w4∑NeighborVoxel+w3∑Fc+w2∑Ec+w1∑Vcw4>w3>w2>w1,branch-voxel=vbranch ∣ costvbranch=max⁡costvcandidate,

where ∑NeighborVoxel means the number of candidates neighbor, ∑*F*_*c*_, ∑*E*_*c*_, and ∑*V*_*c*_ are, respectively, the number of face-connected, edge-connected, and vertex-connected candidates. The voxels of three connected types are also marked in Figure 4. In our implementation, the weighting factors are set as *w*_4_ = 4, *w*_3_ = 3, *w*_2_ = 2, *w*_1_ = 1.

Connecting the topological voxels with the corresponding edges, we can construct an undirected graph to present the geometric structure of vessel system. Based on the graph of topological voxels, it is convenient for us to measure the geometric attributes of vessel system. The measurements are summarized in Table 1.

#### 3.1.4. Tree Generation

To simulate the structure of vasculature and indicate the blood flow, we transform the topological graph of vessel system into vessel trees. First, we convert the undirected graph to a directed one through breadth-first-searching from the vessel root. The root of graph *V*_root_ is the main portal vein, which can be specified as the end-voxel with largest radius summation of it and its branches. It is defined as follows:

(7) Rv=Radiusv+MeanRadiusedgev,Vroot=v0 ∣ Rv0=max⁡Rv,  v∈end-voxel.

**Figure 5:**
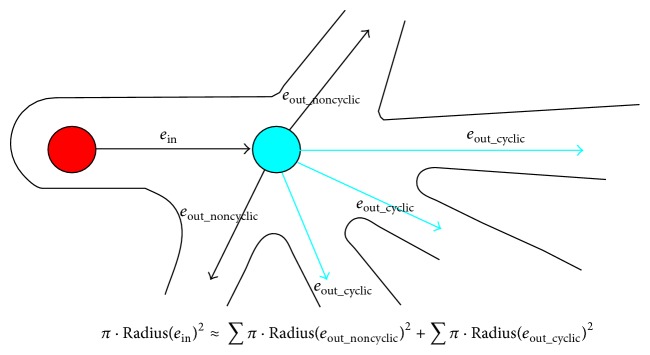
Determining irrelevant vessel branches.

Since the segmented vessel region contains internal cavities, holes, and bays, the generated graph is always cyclic. There are basically two kinds of loops in the graph: redundant branches (with self-loops) and irrelevant branches (with cycles). The redundant branches are easily removed by deleting branches in which all the skeleton voxels share the same nearest topological voxel. Removing irrelevant branches will be a more complex task. According to anatomy theory, at each ramification point, the blood inflow should be equal to outflow. Based on this, we can match the vessels on blood routine and remove the irrelevant ones. Specifically, the outflow of branches should match the inflow of root vein and the blood flow can be approximated with cross-sectional area of veins, which is square of radius.  Figure 5 illustrates a vessel system including one root vein and a branch of five vessels. Among all the connected cyclic edges marked in light blue, we should find a combination set of them that makes outflow most closely match inflow. The vessels out of the combination set are considered as irrelevant branches and should be removed.

The process of determining irrelevant cyclic branches can be formally defined by the following equations:

(8) Diffecomb=Radiusein2−∑Radiuseout_noncyclic2−∑Radiusecomb2,ebranch=arg mine⁡Diffecomb,ecomb⊆eoutcyclic,eirrelevant=e ∣ e∈eout_cyclic∧e∉ebranch,

where *e*_out_cyclic_ = {*e*_out_cyclic_^1^,…, *e*_out_cyclic_^*m*^} consists of all *m* connected cyclic branches and *e*_comb_ denotes a possible combination set of cyclic branches. Diff(*e*_comb_) measure the blood difference between inflow of root vein and outflow of branches. *e*_branch_ is the combination set whose blood flow matches the inflow of root vein. The branches not contained in *e*_branch_ are considered as irrelevant cyclic vessel branches. Through removing the redundant branches and irrelevant branches, vascular tree *T*_vessel_ of portal vessels is generated, which consists of a set of vertexes *V*_vessel_ and directed edges *E*_vessel_ to indicate the blood flow.

### 3.2. Liver Segment and Annotation

#### 3.2.1. Hierarchical Vascular Tree

Considering the blood flow in vasculature, we can further extend the vessel tree to hierarchical vascular tree. As introduced above, vascular tree is formulated by a directed acyclic graph *T*_vessel_ = (*V*_vessel_, *E*_vessel_); the edge direction represents the blood flow. According to blood-supply amount of branches, the vascular tree can be hierarchically divided into two levels. The First Subtree has the branches of large mean radius and generally denotes the main vessel of liver portal vein. Compared with First Subtree, the Second Subtree denotes the branch of smaller mean radius which is widely distributed in liver segments.

**Figure 6:**
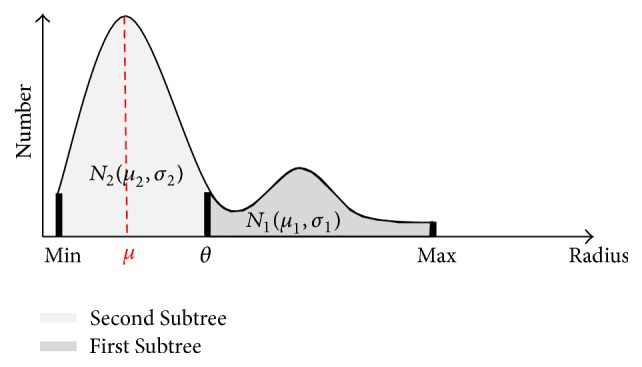
Radius statistics of vessel tree branches.

According to the physiological characteristics of vasculature [33], First Subtree generally consists of limited number of main vessels and Second Subtree involves abundant minor vessels of smaller radius. Based on this, we can categorize vessel trees through modeling the distribution of vessel radius. For implementation, we utilize a mixture of Gaussian distributions (GMM) to approximate the vessel radius distribution.  Figure 6 illustrates the radius statistics of all the vessel tree branches. Min and Max denote the minimum and maximum radius, respectively. Obviously, there are two clusters in the histogram: one centers at small radius and another one locates in the interval of big radius. Having many small branches of similar radius, Second Subtree corresponds to the cluster with higher peak centered at small radius. On the other side, First Subtree is represented by the smaller cluster centered at large radius. Suppose that the radius distributions of two clusters are Gaussian and have forms *N*_2_(*μ*_2_, *σ*_2_) and *N*_1_(*μ*_1_, *σ*_1_), let *μ* = *μ*_2_, the radius range of Second Subtree is [Min, 2*μ* − Min], and the threshold *θ* that separates First and Second Subtree can be computed as *θ* = 2*μ* − Min. For easy implementation, the threshold can be set default as *θ* ≈ 0.5 × Max. In real applications, the threshold can also be online tuned referring to 3D visualization.

Based on the distribution of branch radius, we can determine the subtree of vessels at different levels.

**Definition 6 (First and Second Subtree).**

Given vascular tree *T*_vessel_ = (*V*_vessel_, *E*_vessel_) and threshold *θ*, for each edge *e* ∈ *E*_vessel_, if *θ* < Radius(*e*) ≤ Max, edge *e* belongs to the First Subtree. Otherwise, if Min ≤ Radius(*e*) ≤ *θ*, *e* belongs to the Second Subtree.

As introduced in Section 1, only the connecting branches in Second Subtree will be preserved for the subsequent liver annotation. Among those branches, the Micro Subtree, which represents the trivial structure of vessel system, will be further removed.

**Definition 7 (Micro Subtree).**

Given a tree in Second Subtree, if the number of vertexes in the tree is no more than five, that is |*V*_vessel_| ≤ 5, the tree is considered as Micro Subtree.

The blood flow of Second Subtree actually presents the circulation of vessel system and thereby indicates the structure of functional segments. We use *K*-means++ to cluster the root vertexes of the preserved Second Subtrees and the root clustering will induce a partition of liver region. Each cluster of vessel trees corresponds to a functional segment of liver. Since all the vertexes in the same tree belong to the same blood-supply branch, they are definitely in the same segment. Anatomically, the liver is divided into eight segments according to Couinaud classification. Therefore, the number of the vessel tree clusters is set as *K* = 8. After clustering, we complete the branch division of vascular tree.

#### 3.2.2. Liver Annotation

Based on the branch division of hierarchical vascular tree, the liver voxels are iteratively classified into eight parts using a minimum distance classifier [34]. Let *B* stand for the divided branches in vascular tree, *B*_*i*_; *i* = 1,2,…, 8 is *i*th subtree with vertexes *V*_*B*_*i*__. For each voxel *v* in liver region *I*_liver_, the classifier computes the minimum distance between *v* and *B*_*i*_ to determine which branch supplies blood to *v*, see Definition 8. Through classifying the voxels to different vessel branches, the functional segments of liver are partitioned.

**Definition 8 (Branch Distance).**

For any pair of voxels *v*_liver_ ∈ *I*_liver_ and *v*_branch_ ∈ *B*, Dist(*v*_liver_, *v*_branch_) is the Euclidean distance between the two voxels. Based on the voxel distance, we can define the distance between *v*_liver_ and branch *B*_*i*_ as MinDist(*v*_liver_, *B*_*i*_) = MinDist(*v*_liver_, *v*_branch_). The liver voxel *v*_liver_ will be classified into *k*th branch if *k* = arg min_*i*_⁡(MinDist(*v*_liver_, *B*_*i*_)).

Integrating the functional segments of liver and the organ features obtained from the topological graph of vessels, we can generate the report of liver annotation. The annotation report consists of the functional region visualization and the clinical features to describe the characteristics of liver system. Moreover, the clinical features can be categorized into two groups. Global features mainly include the size of liver, vessels, and lesions, as well as the ratio of each segment to liver. Individual features usually consist of anatomical locations, such as the spatial relationship among vasculature, lesions, and liver, and also the segment in which the lesion resides. The annotation results are helpful for doctors to achieve precise evaluation of liver system and reduce the risk of operation.

## 4. Experimental Results

In the experiments, we expect to validate the effectiveness of the proposed vessel-tree-based liver annotation method. The experiments consist of the tests of vessel tree generation and liver annotation. In the test of vessel tree generation, we verify the vessel skeletonization algorithm and the construction of directed acyclic graph to present the topology of vasculature. In the test of liver annotation, we focus on validating the strategy of partitioning the liver region into functional segments based on hierarchical vascular trees. The experiments are performed on CT dataset stored in format of DICOM images. Each volume has an in-plane resolution of 512 × 512 pixels. The model was implemented based on the toolkits ITK (https://itk.org/) and VTK (http://www.vtk.org/) and was integrated into the MITK framework (http://www.mitk.org/) as a plugin. The computer for program development has an Intel(R) Core(TM)2 Quad CPU (2.66 GHz) and 3.25 GB RAM.

### 4.1. Test of Vessel Tree Generation

**Figure 7:**
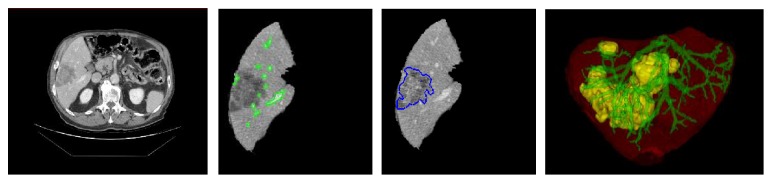
Liver region extraction and visualization.

Applying the improved graph cut model to the treated abdominal CT volumes, we can efficiently produce the reliable segmentation results of liver region. An example is given in Figure 7. The first column is one of the original CT slices. The following two columns show the result of liver region segmentation. The average running time for around 70 slices is about 20 s. More experimental analysis can be found in our previous work [32]. The second and third columns present the segmentation of vessel and tumor in liver region on the same slice. The regions of vessels and tumors are marked in green and blue, respectively. Integrating the segmentation results of a series of CT slices, we can form the 3D visualization of the whole liver region; see the last column. We render the liver region in red, tumors in yellow, and vessels in green (both portal vein and hepatic vein). The visualization indicates that the adopted segmentation method is effective in extracting the liver region from original CT images.

**Figure 8:**
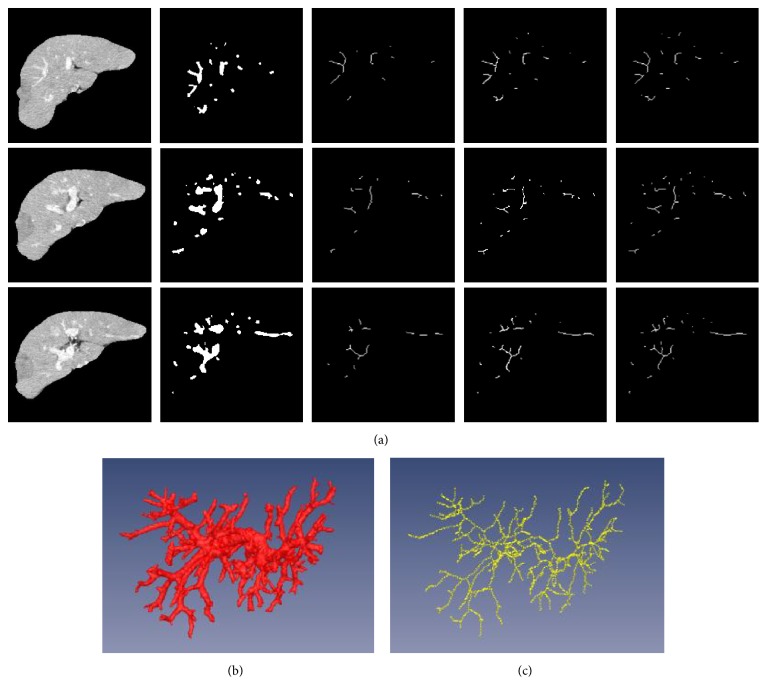
Skeletonization results and visualization.

Based on the segmented vessel regions, we can construct the skeleton and further the topological graph of vasculature. Various kinds of skeletonization algorithms were applied to build up the vessel skeletons, including distance transform algorithms, Voronoi diagram algorithms, and thinning algorithms.  Figure 8(a) shows the vessel skeletons obtained by different skeletonization algorithms. The first column presents three CT images of liver region. The second column illustrates the segmentation of vessel regions. The last three columns show the vessel skeletons generated by distance transform algorithm, Voronoi diagram algorithm, and thinning algorithm, respectively. We find that the thinning algorithm that we use for model implementation can guarantee the connectivity and completeness of the structure of vessel system. Figures 8(b) and 8(c) show 3D visualization of the portal veins and the corresponding skeleton extracted by thinning algorithm.

Compared with the efficiency of different skeletonization algorithms, in 2D space, the average computing time of three algorithms are, respectively, 0.48 s, 0.62 s, and 0.16 s per slice. Taking a CT volume of 124 slices for testing, the computing time of distance transform algorithm is 32.11 s, the thinning algorithm costs 15.79 s, and the Voronoi diagram algorithm runs out of memory. To sum up, the thinning algorithm generates the vessel skeleton in a short time and in the meantime preserves the topology and connectivity of vasculature.

**Figure 9:**
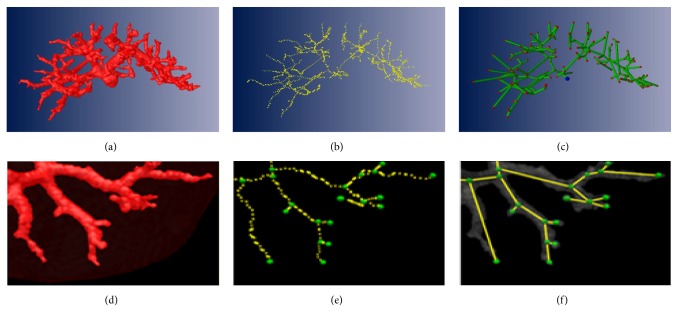
Liver vessel tree generation and visualization.

As introduced in Section 3, with vessel skeletons, we can construct a directed acyclic graph to present the topology of vasculature.  Figure 9 shows the graph representation of the geometric structure of liver portal veins. (a) exhibits the portal veins segmented from liver region. The skeleton result of the portal veins is given in (b). (c) illustrates the directed acyclic graph with the topological voxels marked in red and the tree root marked in blue. Zooming in a local part of liver region, (d) and (e) present the portal veins of local vessel system and its skeleton; (f) shows the induced topological graph. We can find that the proposed method can precisely express the geometric structure of vasculature, even for the minor vessel branches. The time cost for constructing the whole vessel tree is just 1.5 s.

### 4.2. Test of Liver Segment and Annotation

**Figure 10:**
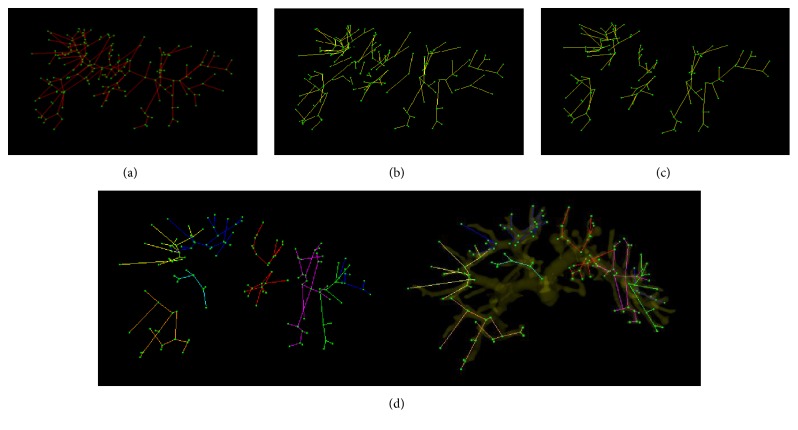
Hierarchical vascular tree generation and division.

**Figure 11:**
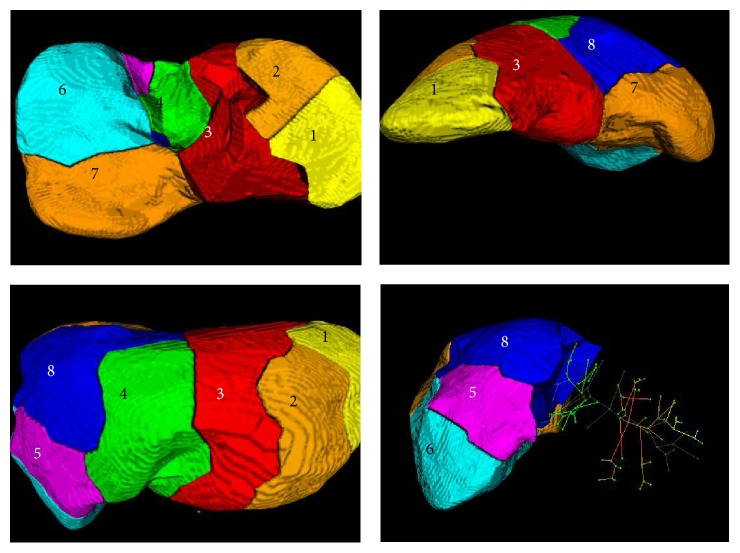
Liver segments and visualization.

According to the blood flow, the vessel trees can be divided into two levels. The First Subtree represents the main vessels of liver portal veins and the Second Subtree denotes the minor branches in vasculature. A liver vessel tree constructed in experiments is shown in Figure 10(a). The tree has 192 vertexes marked in green and 191 edges marked in red. Through measuring the radius of vessels, the branches of the tree are categorized into two groups: 26 branches belonging to First Subtree and 165 branches belonging to Second Subtree. The Second Subtree is shown in Figure 10(b). After removing the Micro Subtree, we obtain the final tree as shown in Figure 10(c). The preserved branches of tree are further clustered into 8 classes using *K*-means++ algorithm.  Figure 10(d) illustrates the clustering results, in which the clusters of branches are marked by different colors. From the view of anatomy, each cluster of vessel branches indicates a functional segment. Thus, through clustering the vessel branches, the liver region can be anatomically divided into eight segments as shown in Figure 11. To achieve complete analysis, the liver segments are exhibited from four different views: in visceral surface, hepatic side, hepatic septal, and right lope. The time cost of the whole process is 5 s.

Based on the functional segments, we can compute the basic organ attributes of liver system, such as voxel number of segment *N*_seg_, segment volume *V*_seg_, volume ratio of segment to liver *R*_seg_, and proportion of tumors in segment *R*_tumor_. Denoting eight functional segments by SegI~SegVIII, the organ attributes of liver segments shown in Figure 11 are listed in Table 2.

Besides basic organ attributes, we can also compute the attributes of liver lopes to support diagnosis.  Table 3 shows the annotation results including the volume information of left/right liver and four liver lopes. It can be inferred from Table 3 that the left lope, which consists of functional segments SegII~SegIV occupies 39.05% of the liver region, and the right lope of segments SegV~SegVIII dominates 55.24%. The statistics are consistent with the anatomical distribution of liver region.

**Figure 12:**
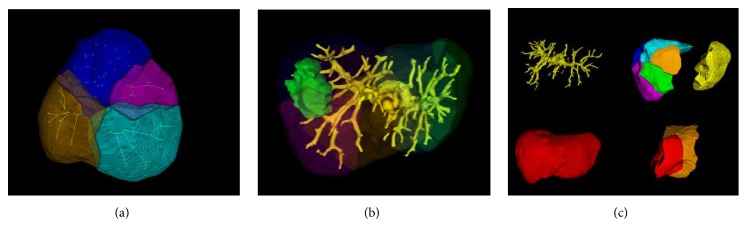
Three-dimensional visualization of liver.

At the final step of the proposed workflow, we should integrate the visualization of liver region, the topological structure of vessel tree, the partition of functional segments, and organ attributes to form a report of liver annotation. As shown in Figure 12(a), 3D visualization of liver region intuitively exhibits the spatial relationship between vasculature, lesions, and liver segments. For example, we can easily observe the blood-supply branches of each segment in right liver lobe.  Figure 12(b) shows the spatial relationship between tumor, portal vein, and functional segments. Moreover, from the visualization results of liver, vasculature and functional segments can be separated, transformed, rotated, and scaled for complete analysis, as shown in Figure 12(c). Abundant experimental results reveal that the proposed vessel-tree-based liver annotation method can provide visual and measurable information for liver system evaluation and thereby it is effective in supporting diagnosis.

## 5. Conclusion

In this paper, we proposed a vessel-tree-based liver annotation method for CT images. At the first step of workflow, the regions of liver, vessels, and tumors are segmented from CT scans. And then a 3D thinning algorithm is applied to obtain the skeleton of liver vessels. Through searching for topological voxels, the skeleton of the portal veins is improved to a directed acyclic graph, that is, vessel tress to present the topology of vasculature. According to the blood flow, the vessel trees are categorized into First and Second Subtrees and the structure of Second Subtrees can indicate the organization of functional segments of liver. In the light of this finding, we cluster the Second Subtrees to partition the liver region into eight functional segments according to Couinaud classification of liver anatomy. Based on the partitioned functional regions, the organ attributes are computed to form quantitative descriptions of liver. At the final step of workflow, we integrate the visualization of liver region, the topological structure of vessel tree, the partition of functional segments, and organ attributes to form a report of liver annotation. Experimental results validate the effectiveness of proposed vessel-tree-based liver annotation method. Our future work will focus on using individual features, such as locational description and shape features, for liver annotation. The liver annotation based on individual organ features is helpful to recognize whether the tumor is benign or malignant.

## Figures and Tables

**Table 1:** Graph attributes description.

Vertex	Coordinate(*v*)	3D coordinate values of each vertex *v*
	Radius(*v*)	The distance from vertex *v* to its nearest surface voxel of *I*_vessel_

Edge	Length(*e*)	The actual length of the branch
	Distance(*e*)	The Euclidean distance between the two vertexes
	MeanRadius(*e*)	The mean radius of the branch: $MeanRadius(e)=\sqrt{Volume(e)/\left(\pi \cdot Length(e)\right)}$
		Volume(*e*) is the voxel numbers in *e*
	Angle(*e*)	The angle from the parent edge to *e*
	No.(*e*)	The edge belonging to which part of vascular system (portal/hepatic vein)

**Table 2:** Liver segments attributes.

	SegI	SegII	SegIII	SegIV	SegV	SegVI	SegVII	SegVIII
*N*_seg_	53062	78287	169190	115116	58760	144737	152095	157323
*V*_seg_ (mL)	53.06	78.29	169.19	115.12	58.76	144.74	152.10	157.32
*R*_seg_ (%)	5.71	8.43	18.22	12.40	6.33	15.59	16.38	16.94
*R*_tumor_ (%)	8.97	0	3.95	0	4.76	0	0	0

**Table 3:** Liver annotation results.

Liver			Ratio (%)	

Caudal lobe	SegI		5.71	

Left lobe	Left lateral lobe	SegII	26.65	39.05
		SegIII		
	Left medial lobe	SegIVa	12.40	
		SegIVb		

Right lobe	Right anterior lobe	SegVIII	23.27	55.24
		SegV		
	Right posterior lobe	SegVII	31.97	
		SegVI		
